# Schwalb and Houben Respond to “The Winding Road to ARTI”

**DOI:** 10.1093/aje/kwad163

**Published:** 2023-07-21

**Authors:** Alvaro Schwalb, Rein M G J Houben

This article is linked to 'Impact of Reversion of *Mycobacterium tuberculosis* Immunoreactivity Tests on the Estimated Annual Risk of Tuberculosis Infection' and 'Invited Commentary: The Winding Road to Identifying the Annual Rate of Tuberculosis Infection' (https://doi.org/10.1093/aje/kwad028 and https://doi.org/10.1093/aje/kwad125).

We appreciate the commentary by Prof. Horsburgh (1) on our article (2), particularly as it stimulates further discussion on where the true annual risk of infection (ARI) of *Mycobacterium tuberculosis* lies. Even though the phenomenon of immunoreactivity reversion is neither new nor rare, its implications for the estimation of the ARI have been largely dismissed. Given how the ARI is calculated, the observed changes in immunoreactivity lead to concrete discrepancies in the metric when accounting for reversion. Our work explored how ARI estimation is affected when accounting for varying reversion probabilities ranging from 0% per year to 50% per year, covering a wide range of scenarios (2). Note that in our paper and this response, we consider reversion the observed process of waning immunoreactivity, rather than an underlying mechanism such as self-cure.

This wide range was chosen to capture the potential impact of past, current, and future tools, reflecting that reversion rates may vary by tool and population and that actual rates are unknown. To anchor our work in data, we overlapped empirical reversion probabilities to visually portray ARI underestimation in a number of selected studies (see Figure 2 in our article (2)).

Criteria for conversion and reversion of tuberculin skin tests, along with the more contemporary interferon-γ release assays, have since been refined (3–5). Specifically to the data in our paper, the study by Grzybowski and Allen (6) in Ontario, Canada (1958–1962) used a low threshold (5 mm) for positivity, and the inherent instability of tuberculin skin testing possibly raised the observed reversion probability, particularly among the younger population (22%/year for persons under 20 years of age). Although Bacillus Calmette-Guérin (BCG) vaccination among children usually leads to more false-positive results (and consequently more reversion), BCG vaccine was not used in Ontario for newborns or infants at the time, so it was unlikely to affect results (6). In the study by Fine et al. (7) from Malawi (1980–1989), more stringent, modern definitions for reversion were used, yet they still resulted in high observed reversion probabilities (ranging from 6%/year to 12.5%/year in persons under 15 years of age) and would result in substantial ARI underestimation, by a factor of 2, from age 12 years onwards (accounting for reversion probabilities in a BCG-naive population) (2). Despite the updated criteria, the Ontario reversion probabilities were chosen over the ones from Malawi because the reported data from the former study allowed us to calculate an uncertainty range.

Prof. Horsburgh suggests another source of data reported by Ferebee (8) as part of the isoniazid trials conducted in the United States. While we were unsure of the precise source for the 10% reversion rate after an interval of 10 years (roughly 1%/year) that was cited, results from the state hospital in Milledgeville, Georgia, in 1968 (Table V in the Ferebee paper (8)) showed a reversion probability of 12.6% in the placebo arm using a 10-mm cutoff and 7.9% using the 5-mm cutoff. Because this was probably an adult population (ages were not provided), a better comparison would perhaps be the results reported among children in Table IV (8). Here, higher reversion probabilities were found, with 50% and 20% of children having reverted at 10 years of follow-up (resulting in approximate rates of 5%/year and 2%/year) using a 5-mm and 10-mm cutoff, respectively, both resulting in a true ARI of up to 1.5 times higher than calculated; this 50% increase already has direct implications (e.g., extrapolation to tuberculosis dynamics or burden).

Therefore, while the annual rate in Ontario was probably a high-end estimate, it is likely that for tuberculin skin testing the reversion rate in children is above 1% per year. Likewise, there is present evidence of high annualized reversion rates of around 5% among adolescents (9); hence, the potential impact of interferon-γ release assay reversion on the estimated ARI (approximately a 2-fold increase) should not be dismissed.

Our study highlights the stark discrepancy between the observed and true ARI when accounting for reversion and, therefore, why we should be cautious in the direct interpretation of the resulting ARI from immunoreactivity surveys (2). Given that children are the primary target population for ARI surveys (10) and studies have repeatedly shown increased reversion rates in this age group (6, 8), it will be essential to quantify and account for reversion.

Here we agree with Prof. Horsburgh in that better and well-characterized data on the rate of reversion are needed to feed into our model (2). Such data should be specific to the tool, age group (particularly for children under age 15 years), and ideally geography. With such data we should also be able to examine quantitatively the suggestion by Prof. Horsburgh that the probability of reversion decreases with time since exposure (1).

While many gaps remain in our understanding of reversion and its impact on ARI underestimation, we hope that this welcome discussion increases recognition of the issue and stimulates appropriate empirical measurements. Better to deal with the known unknowns than to be in the dark overall.
